# Functional requirement of terminal inverted repeats for efficient *ProtoRAG* activity reveals the early evolution of V(D)J recombination

**DOI:** 10.1093/nsr/nwz179

**Published:** 2019-11-13

**Authors:** Xin Tao, Shaochun Yuan, Fan Chen, Xiaoman Gao, Xinli Wang, Wenjuan Yu, Song Liu, Ziwen Huang, Shangwu Chen, Anlong Xu

**Affiliations:** 1 State Key Laboratory of Biocontrol, Guangdong Key Laboratory of Pharmaceutical Functional Genes, School of Life Sciences, Sun Yat-sen University, Guangzhou 510275, China; 2 Laboratory for Marine Biology and Biotechnology, Qingdao National Laboratory for Marine Science and Technology, Qingdao 266237, China; 3 School of Life Sciences, Beijing University of Chinese Medicine, Beijing 100029, China

**Keywords:** *ProtoRAG* transposon, terminal inverted repeat, V(D)J recombination, recombination signal sequence, evolution

## Abstract

The discovery of *ProtoRAG* in amphioxus indicated that vertebrate RAG recombinases originated from an ancient transposon. However, the sequences of *ProtoRAG* terminal inverted repeats (TIRs) were obviously dissimilar to the consensus sequence of mouse 12/23RSS and recombination mediated by ProtoRAG or RAG made them incompatible with each other. Thus, it is difficult to determine whether or how 12/23RSS persisted in the vertebrate RAG system that evolved from the TIRs of ancient RAG transposons. Here, we found that the activity of *ProtoRAG* is highly dependent on its asymmetric 5′TIR and 3′TIR, which are composed of conserved TR1 and TR5 elements and a partially conserved TRsp element of 27/31 bp to separate them. Similar to the requirements for the recombination signal sequences (RSSs) of RAG recombinase, the first CAC in TR1, the three dinucleotides in TR5 and the specific length of the partially conserved TRsp are important for the efficient recombination activity of *ProtoRAG*. In addition, the homologous sequences flanking the signal sequences facilitate ProtoRAG- but not RAG-mediated recombination. In addition to the diverged TIRs, two differentiated functional domains in BbRAG1L were defined to coordinate with the divergence between TIRs and RSSs. One of these is the CTT* domain, which facilitates the specific TIR recognition of the BbRAGL complex, and the other is NBD*, which is responsible for DNA binding and the protein stabilization of the BbRAGL complex. Thus, our findings reveal that the functional requirement for *ProtoRAG* TIRs is similar to that for RSS in RAG-mediated recombination, which not only supports the common origin of *ProtoRAG* TIRs and RSSs from the asymmetric TIRs of ancient RAG transposons, but also reveals the development of RAG and RAG-like machineries during chordate evolution.

## INTRODUCTION

In jawed vertebrates, the adaptive immune system relies on V(D)J recombination to assemble arrays of widely separated variable (V), diversity (D) and joining (J) gene segments to generate hundreds of millions of highly diversified antigen receptors for the recognition of a wide range of pathogens. V(D)J recombination is initiated by the RAG1/RAG2 complex to cleave at the borders of recombination signal sequences (RSSs) adjacent to each V, D and J gene segment [1]. In their consensus sequence, RSSs contain conserved heptamer (5′-CACAGTG-3′) and nonamer (5′-ACAAAAACC-3′) elements separated by two varied spacer sequences of either 12 or 23 bp, which are defined as 12RSS and 23RSS, respectively. Efficient recombination occurs only between a 12RSS and a 23RSS—a restriction known as the 12/23 rule, which ensures the recombination of V–(D)–J in the right order [2].

In 12/23 RSSs, the heptamer, especially the first ‘CAC’ triplet, is critical for RAG complex-mediated DNA cleavage [3]. The crystal structure of the RAG–RSS complex revealed that RAG1 specifically makes contact with the heptamer through multiple domains. Notably, RAG1 contacts the first ‘CAC’ triplet of the heptamer by its helices α16 and α23 [4]. In addition to interacting with the conserved heptamer, RAG1 makes contacts with the nonamer through its NBD domain, which adopts an intertwined dimer structure that mediates the synapsis of two nonamer DNAs and is critical for anchoring RAG proteins onto the RSS [5]. For the spacer sequence, it was initially thought that only the variation in the length of the spacer was deleterious for recombination. However, variations in the spacer sequence were then known to profoundly affect recombination frequency [6,7]. Within the 12RSS and 23RSS spacers, the first five positions contact the RAG complex directly and represent the most conserved consecutive spacer segments within genomes [4]. Overall, not only the consensus sequences of the heptamer and nonamer, but also the length and conservation of the spacers in RSSs can affect the relative representation of gene segments in the Ig and TCR primary repertoires [3,8,9].

Since Tonegawa and colleagues first noticed that the inverted pairing of 12RSS and 23RSS was reminiscent of the TIRs flanking a DNA transposon [10], the host domestication of a RAG-like DNA transposon in the ancestor of jawed vertebrates was proposed for the origin of V(D)J recombination [11]. In the past two decades, several RAG-like proteins and transposons with sequence similarity to RAG1 and RAG2 have been identified [12–19]. The discovery of *ProtoRAG* in the lancelet (*Branchiostoma belcheri*), a basal chordate, mechanistically linked the RAG transposon to the vertebrate RAG machinery [20].

The typical *ProtoRAG* transposon in lancelets contains a pair of tail-to-tail-oriented RAG1-like and RAG2-like genes (referred to as *BbRAG1L* and *BbRAG2L*), which are flanked by 5-bp target site duplications (TSDs) and a pair of TIRs [20]. Except for the presence of an additional repeat region in BbRAG1L and the lack of a PHD domain in BbRAG2L, both BbRAG1L and BbRAG2L contain similar conserved domain architectures as vertebrate RAG proteins, including the conserved core regions. The BbRAG1L/BbRAG2L proteins encoded by *ProtoRAG* can mediate TIR-dependent transposon excision, host DNA joint (HDJ) recombination, transposition and even TIR signal joint formation at a low frequency, as is the case for jawed-vertebrate RAGs [20]. Recently, with the help of the cryo-electron microscopy structure of the BbRAG–TIR complex, jawed-vertebrate-specific adaptations in RAG recombinase were revealed to have been involved in the host domestication of ancestral RAG transposons [21]. However, unlike the obvious conservation between RAG(-like) proteins during evolution, the paired TIRs in *ProtoRAG* from lancelets show limited sequence identity to the 12/23RSS [20]. Notably, BbRAG1L and BbRAG2L can act on TIRs but not on RSSs, while the opposite is observed for mouse RAG, which can act on RSSs but not TIRs from *ProtoRAG* [20]. Sequence divergence is also observed in other TIRs of RAG-like transposons (Supplementary Fig. 1), such as the pairs of 5′/3′TIRs from the RAG-like transposons in the sea star (*Patiria miniata*) and acorn worm (*Ptychodera flava*) [12,19]. Even the comparison of RSS sequences from zebrafish and humans revealed the presence of species-specific features [22]. Moreover, most *Transib* families are flanked by symmetric TIRs, such as the *Hztransib* TIRs, which have clear sequence similarity to the RSS heptamer but little or no sequence similarity to the RSS nonamer [15,18,23]. Thus, whether or how 12/23RSS persisted in the vertebrate RAG system by evolving from a paired TIR in an ancient RAG transposon is difficult to determine.

Herein, we performed a comprehensive study to explore the functional requirements of TIRs for *ProtoRAG* to mediate efficient DNA recombination and found that the requirements of TIRs according to *ProtoRAG* are paralleled by those of RSSs according to RAG recombinase in spite of the sequence divergence between them. Thus, we implied that both the *ProtoRAG* and vertebrate RAG system (RAG recombinase and 12/23RSS) evolved from an ancient RAG transposon with paired asymmetric TIRs.

## RESULTS

### Characterization of the functional TIRs required for efficient *ProtoRAG* activity

**Figure 1.**
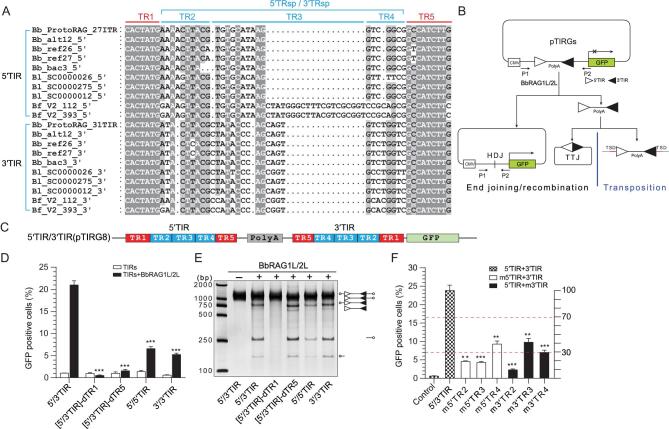
Characterization of the functional TIRs required for efficient *ProtoRAG* activity. (A) Alignment of *ProtoRAG* 5′TIR and 3′TIR sequences from the three species of lancelets. Shading indicates sequence conservation, with a darker gray indicating an increased degree of conservation. The common structure of the TIRs was defined as indicated above the alignments. Bb, *B. belcheri*; Bf, *B. floridae*; Bl, *B. lancealatum.* The *ProtoRAG* copy identification numbers correspond to their insertion scaffolds. (B) Schematic diagrams of the recombination assay and PCR assay used to measure BbRAG1L/2L-mediated DNA excision and recombination. After recombination, GFP was expressed in the cells and quantification of the GFP-positive cells was performed by flow cytometry; the HDJ product was detected by PCR. Unfilled and filled triangles, 5′TIR and 3′TIR sequences of *ProtoRAG*, respectively; P1/P2, PCR primers. (C) Composition diagram of the wide-type 5′/3′TIR substrate. A mini-transposon composed of 5′TIR, reversed 3′TIR and a separating PolyA sequence was inserted upstream of *GFP* in the pEGFP-N1 plasmid to control its expression and was named pTIRG8. (D) Quantification of the GFP-positive cells produced by BbRAG1L/2L-mediated recombination with several altered TIR substrates. *BbRAG1L/2L* was co-transfected with distinct TIR substrates into HEK293T cells. 5′/3′TIR: wide-type 5′/3′TIR substrate with the composition shown in (C); [5′/3′TIR]-dTR1: with deletion of the TR1 elements from both the 5′TIR and 3′TIR, where -d means deletion; [5′/3′TIR]-dTR5: with deletion of the TR5 elements from both the 5′TIR and 3′TIR; 5′/5′TIR: substrate with two paired 5′TIRs; 3′/3′TIR: substrate with two paired 3′TIRs. (E) Cleavage of distinct TIR substrates by BbRAG1L/2L *in vitro*. The composition of the cleavage product is shown on the left according to the length of the corresponding fragments. Unfilled and filled triangles, 5′TIR and 3′TIR of *ProtoRAG*, respectively. (F) Quantification of the GFP-positive cells produced by BbRAG1L/2L-mediated recombination with several TRsp-mutated substrates. The *x*-axis shows the TIR substrates, the left *y*-axis shows the percentage of GFP-positive cells and the right *y*-axis shows the percentage of GFP-positive cells relative to the value of 5′/3′TIR. Three effect levels (slight, moderate, dramatic) were defined according to the value of the right *y*-axis (>70%, 30%–70% or < 30%). m5′TR2: only 5′TR2 in 5′/3′TIR was mutated, with a normal 3′TIR; m3′TR2: only 3′TR2 in 5′/3′TIR was mutated, with a normal 5′TIR; other substrates were named according to the same nomenclature. m- means mutation by replacement with irrelevant nucleotides. Control: represents the background value of 5′/3′TIR (pTIRG8) without expression of BbRAG1L/2L (same in all recombination assays). The number of GFP-positive cells is expressed as the mean (+/– SEM) and significant differences were analyzed with a two-tailed Student's *t*-test after comparing the number of GFP-positive cells with those of 5′/3′TIR; the significance levels are indicated according to the *p*-values: *: *p* < 0.05, **: *p* < 0.01, ***: *p* < 0.001.

Our previous study revealed that the activity of *ProtoRAG* depends on its 5′TIR and 3′TIR [20]. Here, to further reveal the typical features of *ProtoRAG* TIRs, the TIRs in several *ProtoRAG* copies from three species of lancelets (*Branchiostoma belcheri*, *Branchiostoma lancealatum* and *Branchiostoma floridae*) were used for sequence alignments (Fig. 1A). The terminal 7 bp (5′-CACTATG-3′) resembles the consensus RSS heptamer (5′-CACAGTG-3′), which is identical in all *ProtoRAG* TIRs and is referred to as TR1. Another highly conserved 9-bp block existing at the 3′ end of all *ProtoRAG* TIRs was identified and is referred to as TR5. Similar to RSSs, TR1 and TR5 in the 5′TIR and 3′TIR of the *B. belcheri ProtoRAG* are separated by additional 27- and 31-bp elements, which are referred to as 5′TRsp and 3′TRsp, respectively. Both 5′TRsp and 3′TRsp include a partially conserved 9- to 10-bp element adjacent to TR1 and two varied blocks adjacent to TR5, referred to as TR2, TR3 and TR4, respectively (Fig. 1A). The full-length 5′TIR and 3′TIR (including TR1–TR5) can be efficiently recognized by co-expressed BbRAG1L/BbRAG2L complexes but not singly expressed BbRAG1L or BbRAG2L complexes (Supplementary Fig. 2A and B), which is consistent with our previous observation that BbRAG1L must cooperate with BbRAG2L to cleave the TIR substrates (based on new data presented as Supplementary Fig. 2C) [20].

To further define the elements within 5′/3′TIRs essential for the function of *ProtoRAG*, a series of mutated TIR substrates were generated for *ex vivo* recombination assays and *in vitro* cleavage assays (Fig. 1B and C). The full deletion of TR1 within the 5′/3′TIR eliminated substrate recombination *ex vivo* and substrate cleavage *in vitro* by the BbRAG1L/BbRAG2L complex (Fig. 1D and E), indicating that TR1 may play a critical role in the initiation of substrate cleavage. The full deletion of TR5 within the 5′/3′TIR had a deleterious effect on recombination *ex vivo* but showed little effect on substrate cleavage *in vitro*, except that it resulted in the generation of a slightly more unspecific cleavage product (Fig. 1D and E). In addition, the replacement of paired 5′/3′TIR with 5′/5′TIR or 3′/3′TIR as a substrate moderately decreased the recombination efficiency *ex vivo.* Decreased cleavage when using 5′/5′TIR but not 3′/3′TIR as a substrate was also observed (Fig. 1D and E). These results indicate that a pair of heterologous 5′/3′TIRs is preferred by ProtoRAG for mediating recombination *ex vivo*. Furthermore, substitution of the TR2, TR3 and TR4 elements in either 5′TIR or 3′TIR resulted in decreased recombination (Fig. 1F), which emphasizes the importance of the sequences in both TRsps in the 5′/3′TIRs for functional TIRs.

In short, 5′TIR and 3′TIR in *ProtoRAG* from *B. belcheri* presented a typical structure composed of conserved TR1 and TR5 elements and the partially conserved separating element TRsp (27 bp in 5′TIR and 31 bp in 3′TIR, respectively). Although these elements seem to play different roles according to the different effects of their mutation, the whole sequences of 5′TIR and 3′TIR are important for functional TIRs and the induction of efficient *ProtoRAG* activity.

### The first CAC triplet in 5′/3′TIRs is essential for the activity of *ProtoRAG*

**Figure 2.**
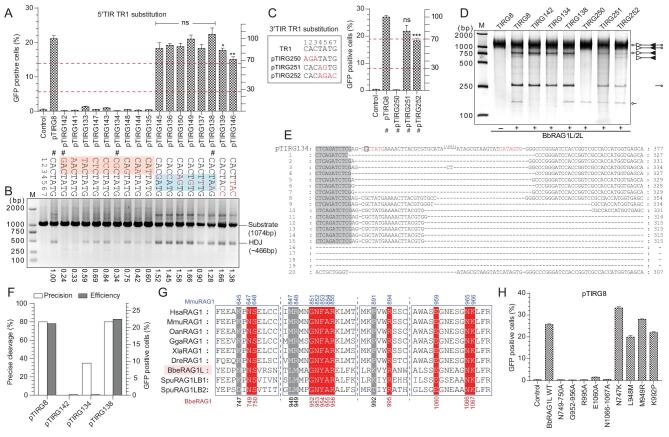
Recombination and cleavage assays used to reveal the critical nucleotides in the TR1 element. (A) Quantification of GFP-positive cells produced by BbRAG1L/2L-mediated recombination with the 5′TR1-altered substrates. The position in 5′TR1 was assigned and the substituted nucleotides are shown as indicated (marked in red). The substrates chosen for the cleavage assay are marked by #, and the left and right *y*-axes are the same as those in Fig. 1F. The red-shaded regions contain the critical nucleotides and the blue-shaded regions denote the changeable nucleotides. (B) PCR assay to detect the recombined HDJ product produced by BbRAG1L/2L-mediated recombination with the corresponding substrates in (A). The value at the bottom shows the amount of HDJ product as quantified with ImageJ using the product of pTIRG8 as the reference. (C) Quantification of GFP-positive cells produced by BbRAG1L/2L-mediated recombination with the 3′TR1-altered substrates. The substitution of nucleotides in the substrates is shown on the left (marked with red color). (D) Cleavage of altered TIR substrates with purified BbRAG1L/2L proteins; the composition of the cleavage product is shown on the right according to the length of the corresponding fragments. Unfilled and filled triangles indicate the 5′TIR and 3′TIR sequences of *ProtoRAG*, respectively. (E) Alignment of the HDJ sequences produced by the recombination of the pTIRG134 substrate. The first line shows the original sequence of pTIRG134, with the TR1 colored red and the A to G mutation marked with a square. HDJ sequences from 20 clones were analysed. The cleavage of the plasmid backbone caused long nucleotide deletions, which are marked with a dash. (F) Statistics of precise cleavage and the recombination efficiency produced by the recombination of several 5′TR1-mutated substrates. The *x*-axis refers to the TIR substrates and the HDJ sequences are shown in (B). The left *y*-axis shows the percentage of precise cleavage and the right *y*-axis shows the averaged percentage of GFP-positive cells. The deletion of fewer than 10 bases in the HDJ sequence with complete transposon sequence removal was considered to indicate precise cleavage. (G) Multiple sequence alignment of RAG1 proteins from multiple species. The sites that may be responsible for CAC nucleotide contacts are shown. The conserved sites are shaded in red and the ambiguous sites are shaded in gray. The positions of these amino acids in BbRAG1L are indicated at the bottom and at the top for mRAG1. The abbreviations of the species: Hsa, *Homo sapiens* (humans); Mmu, *Mus musculus* (mouse); Oan, *Ornithorhynchus anatinus* (platypus); Gga, *Gallus gallus* (chicken); Xla, *Xenopus laevis* (frog); Dre, *Danio rerio* (zebrafish); Bbe, *Branchiostoma belcheri* (amphioxus). (H) Quantification of GFP-positive cells produced by mutated *BbRAG1L* together with the *BbRAG2L* and pTIRG8 substrates by flow cytometry. The numbers of GFP-positive cells are expressed as the mean (+/– SEM) and significant differences were analysed with a two-tailed Student's *t*-test after comparing the number of GFP-positive cells with those in pTIRG8. The significance levels are indicated according to the *p*-values: *: *p* < 0.05, **: *p* < 0.01, ***: *p* < 0.001.

The TR1 element (5′-CACTATG-3′) in 5′/3′TIRs shows great resemblance to the consensus RSS heptamer (5′-CACAGTG-3′) in vertebrates, and it can be bound by the BbRAGL complex. To further determine which nucleotides within TR1 are critical for the function of *ProtoRAG*, a series of mutated substrates was constructed for *in vitro* cleavage and *ex vivo* recombination assays. The results of the *ex vivo* recombination assays showed that any substitutions within the first CAC triplet of TR1 in 5′TIR could completely eliminate BbRAGL-mediated recombination (Fig. 2A). Substitutions of nucleotides at positions 4–6 had no significant effect on recombination, but multiple substitutions, including that of G at position 7 (pTIRG139 and pTIRG146), moderately decreased the recombination efficiency (Fig. 2A). Notably, the substrate pTIRG150, which contains the same sequence as the RSS heptamer consensus sequence in its 5′TR1 element, produced a recombination efficiency comparable to that of the substrate with a normal 5′TIR. Changes in the recombination activities of these altered substrates were also reconfirmed by PCR by detecting the HDJ (Fig. 2B). Moreover, HDJs from several typically mutated substrates were recovered and ligated into vectors for sequencing. Precise cleavage in mutated TIR substrates was greatly disrupted by changing the first CAC triplet in TR1 into GAC or CGC (pTIRG134 in Fig. 2E and F, pTIRG8 in Supplementary Fig. 3A). However, no difference was observed for the substitution of the 6th base in TR1 (pTIRG138 in Fig. 2F and Supplementary Fig. 3B). The results of the substitution of TR1 in 3′TIR also confirmed the importance of the first CAC triplet and the last G for the function of *ProtoRAG* (Fig. 2C).

Consistently, the substitution of CAC in the TR1 of 5′TIR completely impaired the cleavage of 5′TIR but did not alter the cleavage of normal 3′TIR (TIRG142, TIRG134 in Fig. 2D). Additionally, the CAC mutation in 3′TIR resulted in impaired cleavage of 3′TIR but not of normal 5′TIR (TIRG250 in Fig. 2D) and substitutions at positions 4–7 in TR1 slightly affected cleavage of 3′TIR (pTIRG251 and TIRG252 in Fig. 2D). These results indicate that the first CAC triplet in TR1 in both 5′TIR and 3′TIR is critical for the activity of *ProtoRAG* and the other four bases are not stringently required, except for a preference for ‘G’ at position 7.

The first ‘CAC’ in RSSs were considered as the most conserved nucleotides, which are shared by TIRs in many RAG-like transposons (Supplementary Fig. 1) [3,15]. The conservation of ‘CAC’ was considered to build on their propensity to unwind, which was revealed to be critical for the nicking step of RAG recombinase [24]. In addition, CAC conservation may also be enforced by important contacts with some conserved amino acids in RAG recombinase [25]. By building a homologous model of BbRAG1L according to the structure of the RAG–RSS complex, several sites in BbRAG1L that may be responsible for CAC binding were identified and mutated (Fig. 2G and Supplementary Fig. 3C). The recombination efficiency was dramatically decreased when the conserved sites were mutated in BbRAG1L proteins (red-colored sites in Fig. 2G and H), but the mutations of ambiguous sites had only a weak effect on recombination (gray-colored sites in Fig. 2G and H). These results indicated that the CAC triplet is critical for recombination and that CAC binding was highly conserved in BbRAG1L transposase and RAG1 recombinase during evolution.

### Essential function of TR5 in 5′/3′TIRs in ProtoRAG-mediated recombination

**Figure 3.**
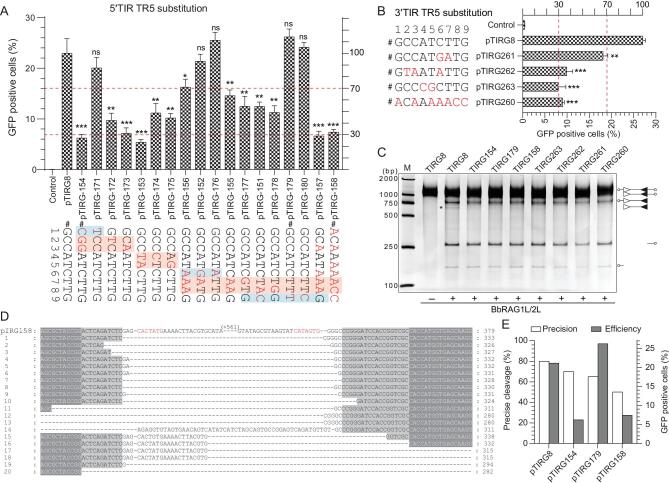
Recombination and cleavage assays to reveal the critical nucleotides in the TR5 element. (A) Quantification of GFP-positive cells produced by BbRAG1L/2L-mediated recombination with the 5′TR5-altered substrates. The position in the 5′TR5 was assigned as indicated. The nucleotide substitutions are shown as indicated (marked in red). The substrates chosen for the cleavage assay are marked by #, and the left and right *y*-axes are the same as those in Fig. 1F. The regions shaded red contain the critical nucleotides and the regions shaded blue denote the changeable nucleotides. (B) Quantification of GFP-positive cells produced by BbRAG1L/2L-mediated recombination with the 3′TR5-altered substrates. The substitutions of the substrate nucleotides are shown on the left (marked in red). (C) Cleavage of altered TIR substrates with purified BbRAG1L/2L proteins; the composition of the cleavage product is shown on the right according to the length of the corresponding fragments. Unfilled and filled triangles indicate the 5′TIR and 3′TIR sequences of *ProtoRAG*, respectively. (D) Alignment of the HDJ sequences produced by recombination with the pTIRG158 substrate. The first line is the original sequence of pTIRG158, with TR1 colored red. HDJ sequences from 20 clones were analysed. Cleavage of the plasmid backbone caused long nucleotide deletions, which are marked with a dash. (E) Statistics for precise cleavage and the average recombination efficiency produced by recombination with several 5′TR5-mutated substrates. The *x*-axis shows the TIR substrates and the HDJ sequences were from HDJ detections in Supplementary Fig. 4A. The left *y*-axis shows the percentage of precise cleavage and the right *y*-axis shows the percentage of GFP-positive cells. The deletion of fewer than 10 bases in the HDJ sequence with complete transposon sequence removal was considered a product of precise cleavage. GFP-positive cells are expressed as the mean (+/– SEM) and significant differences were analysed with a two-tailed Student's *t*-test after comparing the number of GFP-positive cells with those in pTIRG8. The significance levels are indicated according to the *p*-values: *: *p* < 0.05, **: *p* < 0.01, ***: *p* < 0.001.

In V(D)J recombination, the nonamer in RSS is a major binding target of RAG1 through the NBD domain [4,26]. Although the TR5 in 5′/3′TIRs (5′-GCCATCTTG-3′) of *ProtoRAG* is significantly different from the A-rich RSS nonamer (Supplementary Fig. 1), as one of the two conserved elements in 5′/3′TIRs of *ProtoRAG* from three lancelet species, TR5 may play a role in *ProtoRAG* activity *ex vivo*. To reveal the function of the TR5 consensus sequence in *ProtoRAG* TIRs, a series of substrates containing mutated TR5 were prepared and subjected to BbRAGL complex-mediated recombination assays (Fig. 3A and B). Single base substitutions at positions 1, 6 and 9 did not significantly perturb recombination (pTIRG171, pTIRG176 and pTIRG180 in Fig. 3A). The substitution of a C–C dinucleotide at positions 2–3 and an A–T dinucleotide at positions 4–5 resulted in a nearly 70% decrease in the recombination efficiency (Fig. 3A) and the substitution of a T–T dinucleotide at positions 7–8 with A–A moderately decreased the recombination efficiency (pTIRG155 in Fig. 3A). Similar results were observed when the 3′TIR TR5 was mutated (Fig. 3B). Among these mutated substrates, substitutions that produced the same sequences as those of the RSS nonamer consensus in vertebrates resulted in a nearly 70% decrease in the recombination efficiency when compared with that of normal TR5 (pTIRG158 in Fig. 3A and pTIRG260 in Fig. 3B). These results indicated that the nucleotides at positions 2–5 and 7–8 in conserved TR5 elements are critical for the function of 5′/3′TIRs to induce efficient *ProtoRAG* activity.

Compared to the obvious decrease in the recombination efficiency caused by the TR5 mutation, the cleavage of TR5-mutated substrates *in vitro* showed a slight decrease in double-cut products of TIR substrates when critical nucleotides in TR5 were mutated (Fig. 3C). To explore how the TR5 mutations caused an obvious decrease in the recombination efficiency *ex vivo*, the *ex vivo* recombination products were analysed through HDJ detection and sequencing (Supplementary Fig. 4A). It was found that the critical nucleotide mutations in TR5 were deleterious to the formation of precisely rearranged products (Fig. 3D and E). As shown by the alignment of the pTIRG158 HDJ products, imprecise cleavage and mistargeted cleavage could be observed

(clones 15–18) when TR5 within 5′TIR was completely replaced with the RSS nonamer (Fig. 3D). These results indicate that TR5 may play a role in facilitating the precise cleavage of TIR targets by ProtoRAG.

### The length and consensus sequence of TRsp in 5′/3′TIRs is essential for BbRAG1L/2L-mediated DNA recombination

**Figure 4.**
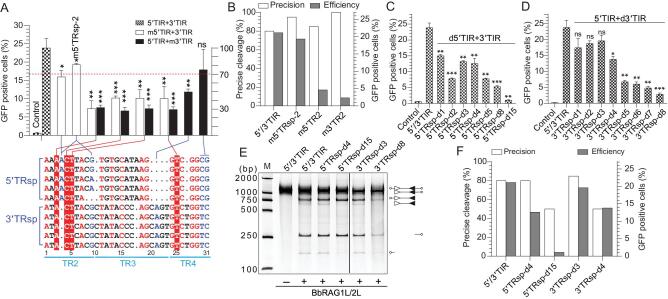
Recombination and cleavage assays revealed the importance of the length and sequence of TRsp for the activity of *ProtoRAG.* (A) Quantification of GFP-positive cells produced by BbRAG1L/2L-mediated recombination with the TRsp-altered substrates. (Below) Alignment of the TRsp sequences from several 5′/3′TIRs of *ProtoRAG* in *B. belcheri*. The conserved nucleotides are colored red and some of them were singly mutated in 5′TIR and 3′TIR (shaded with red). Several non-conserved nucleotides are colored blue and were mutated in 5′TIR or 3′TIR. The regions and the sequence numbers of 3′TIRs are shown as indicated. Recombination corresponding to the mutated substrate in 5′TIR and 3′TIR is shown by the linked lines above the alignment. (Top) The left and right *y*-axes are the same as those in Fig. 1F. (B) Statistics of precise cleavage and the average recombination efficiency produced by recombination with several TRsp-mutated substrates. The left *y*-axis shows the percentage of precise cleavage and the right *y*-axis shows the percentage of GFP-positive cells. The deletion of fewer than 10 bases in the HDJ sequence with complete transposon sequence removal was considered a product of precise cleavage. (C) Quantification of GFP-positive cells produced by BbRAG1L/2L-mediated recombination with partial deletion of the 5′TRsp substrates. All deletions in the substrates began from the 3′-end of 5′TRsp that adjoined 5′TR5 and the length of the deleted nucleotide is shown in the substrate name. The 3′TIR was untouched in these deletions. -d: deletion. (D) Quantification of GFP-positive cells produced by BbRAG1L/2L-mediated recombination with the partial deletion of the 3′TRsp substrates. All deletions in substrates began from the 3′-end of 3′TRsp that adjoined 3′TR5 and the length of deleted nucleotide is shown in the substrate name. The 5′TIR was untouched in these deletions. -d: deletion. (E) Cleavage of the partially deleted TRsp substrates by purified BbRAG1L/2L proteins; the composition of the cleavage product is shown on the right according to the length of the corresponding fragments. Unfilled and filled triangles indicate the 5′TIR and 3′TIR sequences of *ProtoRAG*, respectively. (F) Statistics of precise cleavage and the recombination efficiency produced by recombination with the partially deleted TRsp substrates. The left *y*-axis shows the percentage of precise cleavage and the right *y*-axis shows the percentage of GFP-positive cells. The deletion of fewer than 10 bases in the HDJ sequence with complete transposon sequence removal was considered a product of precise cleavage. GFP-positive cells are expressed as the mean (+/– SEM) and significant differences were analysed with a two-tailed Student's *t*-test after comparing the number of GFP-positive cells with those in 5′/3′TIR. The significance levels are indicated according to the *p*-values: *: *p* < 0.05, **: *p* < 0.01, ***: *p* < 0.001.

To mediate efficient recombination, ProtoRAG prefers the heterologous pair of 5′/3′TIR (Fig. 1F). Since 5′TRsp and 3′TRsp have an overall identity of 45.5% (Fig. 4A), to further reveal whether the lengths of TRsp and the conserved nucleotides in TRsp are essential for efficient *ProtoRAG* activity, a series of mutated substrates with base substitutions or shortened lengths were constructed for the functional analysis. The mutation of several conserved nucleotides in 5′TRsp and 3′TRsp, mainly in the TR2 region, caused an obvious decrease in recombination (red background in Fig. 4A), while the mutation of several non-conserved nucleotides caused a moderate decrease in recombination (blue text in Fig. 4A). However, more extensive mutation of 5′TRsp and 3′TRsp revealed that most of the single nucleotide mutations in TRsp caused a moderate decrease in recombination efficiency (Supplementary Fig. 5A and B). In addition, although the mutation of TRsp may have caused a severe decrease in recombination, mutation did not impair the precision of TIR cleavage (Fig. 4B). These results indicate that, except for some critical nucleotides in TRsp, most of the nucleotides in TRsp allowed low-level variations.

TRsp in TIRs links the conserved TR1 and TR5 elements via a specific number of nucleotides; thus, the shortening of TRsp could remove the nucleotides in TRsp to produce an equivalent mutation to that of the TRsp and TR5 elements in the same time. In the shortened 5′TRsp, the deletion of 1 or 3–4 bases adjacent to the 5′TR5 caused a moderate decrease in recombination efficiency, but the deletion of 2, 5 or 8 bases decreased the recombination efficiency by up to 70% (Fig. 4C). Importantly, when the 5′TRsp within 5′TIR was shortened from 27 to 12 bp, BbRAGL-mediated recombination was almost completely eliminated (5′TRsp-d15 in Fig. 4C). The *in vitro* cleavage assays confirmed that the substrate with a 4-bp deletion in 5′TRsp could be cleaved similarly to normal 5′TIR (5′TRsp-d4 in Fig. 4E), but the substrate with a 15-bp deletion in 5′TRsp resulted in inefficient cleavage of the shortened 5′TIR but normal cleavage of the 3′TIR (5′TRsp-d15 in Fig. 4E). Like the shortened 5′TRsp, the deletion of 1–4 bp of 3′TRsp adjacent to the 3′TR5 modestly decreased the recombination efficiency, but the deletion of 5–8 bp dramatically decreased the recombination efficiency (Fig. 4D). Notably, 3′TRsp-d8 with the same length as the 23RSS spacer in vertebrates resulted in highly inefficient recombination (Fig. 4D). *In vitro* cleavage assays confirmed that the substrate with a 3-bp deletion in 3′TRsp could be cleaved normally (3′TRsp-d3 in Fig. 4E), but the substrate with an 8-base deletion resulted in inefficient cleavage at the site of the shortened 3′TIR (3′TRsp-d8 in Fig. 4E). Previously, the TR5 mutation could decrease the cleavage precision of TIRs by ProtoRAG; here, the shortened TRsp also resulted in decreased precision of the cleavage of TIRs by ProtoRAG (Fig. 4F and Supplementary Fig. 4D), as revealed by the sequencing of the HDJ products of several shortened TRsp substrates (Supplementary Fig. 4B and C). In the alignment of the 3′TRsp-d4 HDJ products, it was shown that imprecise cleavage mainly occurred adjacent to the shortened 3′TIR, but the wide-type 5′TIR was precisely cleaved (Supplementary Fig. 4 G). Considering that nucleotide substitution alone in TRsp did not impair the precision of the cleavage of TIRs, the decreased precision of the cleavage of TIRs in shortened TRsp substrates could be attributed to the displacement of TR5 in TIRs by the shortened TRsp. Taken together, these observations indicate that not only were the critical nucleotides in TRsp in 5′/3′TIR essential for the efficient recombination of *ProtoRAG*, but also that the rigid length of TRsp was critical to keeping a suitable distance between the separated TR1 and TR5 elements to ensure that functional 5′/3′TIRs could induce efficient and precise *ProtoRAG* activity.

### Homologous flanking sequences of TIRs can benefit ProtoRAG-mediated recombination

**Figure 5.**
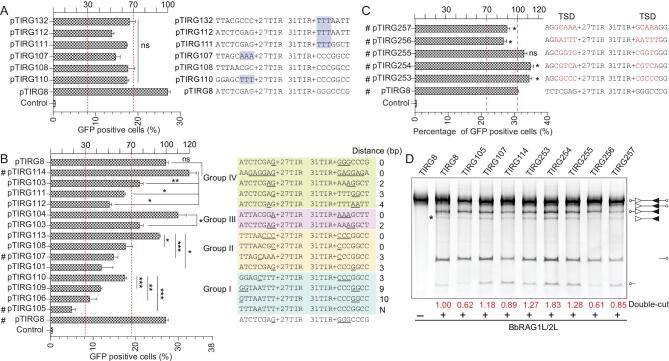
CG-rich microhomology in the flanking sequences is important for the activity of *ProtoRAG*, as revealed by the recombination and cleavage assays. (A) Quantification of GFP-positive cells produced by BbRAG1L/2L-mediated recombination with the TIR substrates containing the altered flanking sequences. AAA and TTT are shaded in blue. Significant differences in GFP-positive cells among the altered substrates were analysed with one-way ANOVA. (B) Quantification of GFP-positive cells produced by BbRAG1L/2L-mediated recombination with TIR substrates containing a homologous flanking sequence. The TIR substrates were divided into four groups according to their flanking sequences; the potential microhomology sequences are underlined and the distance between two microhomology sequences is shown on the right. (C) Quantification of GFP-positive cells produced by BbRAG1L/2L-mediated recombination with TSD sequences containing TIR substrates. TSDs are listed and marked in red. (D) Cleavage of flanking sequence-altered TIR substrates with purified BbRAG1L/2L proteins; the composition of the cleavage product is shown on the right according to the length of the corresponding fragments. Unfilled and filled triangles indicate the 5′TIR and 3′TIR sequences of *Proto*RAG, respectively. The double-cut products (marked with *) were quantified with ImageJ using the product of pTIRG8 as a reference. GFP-positive cells are expressed as the mean (+/– SEM) and significant differences were analysed with a two-tailed Student's *t*-test; the significance levels were indicated according to the *p*-values: *: *p* < 0.05, **: *p* < 0.01, ***: *p* < 0.001.

The transposition of *ProtoRAG* in the genome always causes sequence duplication at the insertion site to form a pair of 5-bp TSDs flanking their TIRs, which exhibit a bias toward GC bases [20]. This base preference was also observed for *Transib* and vertebrate *RAG* [15,27]. In mice, TTT-heptamer-12 spacer substantially impaired the efficiency of V(D)J recombination, and T-heptamer and AAA-heptamer also significantly diminished recombination [28]. The crystal structure revealed that there was substantial interaction between the flanking sequences and the RAG complex, especially for the first 5 bp adjacent to the heptamer [25]. Consistently, the interaction of the BbRAGL complex with 5′TIR was also severely decreased by the deletion of the flanking sequence of 5′TIR (Supplementary Fig. 6A and B). Thus, to explore the roles of the flanking sequence in BbRAGL-mediated recombination, a series of substrates with different flanking sequences were constructed. No significant difference in recombination efficiency was found when TTT, AAA or CCC was adjacent to 5′TIR or 3′TIR (Fig. 5A). Considering that 5′/3′TIRs are flanked by TSDs in *ProtoRAG*, we hypothesized that the homologous sequences of the flanking regions might play roles in the transposition of *ProtoRAG*. Thus, another series of constructs were made to test the effects of homologous flanking sequences on BbRAGL-mediated recombination. An increase in the distance between the first homologous nucleotide, such as in the substrates in groups I and IV, led to reduction of recombination efficiency (Fig. 5B). For example, the construct pTIRG105, which contained no homologous bases in the two flanking sequences, resulted in 80% reduction compared with pTIRG8. Similarly, the substrate pTIRG114, with six homologous bases between its flanking regions, produced efficient recombination (Fig. 5B). *In vitro* cleavage assays using the substrate TIRG105 also showed reduced double-cut cleavage, while TIRG114, with six homologous bases, showed efficient double-cut cleavage (Fig. 5D). Similar results for groups II and III indicated that the microhomology in the two flanking sequences was important for ProtoRAG-mediated recombination (Fig. 5B).

To further verify this observation, five TSDs were inserted into the flanking sequence adjacent to TIRs to form new substrates. The results showed that the new substrates with GC-rich TSDs, such as pTIRG253 and pTIRG254, produced higher recombination efficiencies than the pTIRG8 construct based on *ex vivo* recombination assays (Fig. 5C and Supplementary Fig. 6C). Although constructs with AT-rich TSDs were not as efficient as those containing GC-rich TSDs (pTIRG256 and pTIRG254 in Fig. 5C and Supplementary Fig. 6C), this benefited recombination compared with pTIRG105 and pTIRG107 (Fig. 5B). Consistently, the substrates with CG-rich TSDs (TIRG253, 254, 255 in Fig. 5D) produced more double-cut products in *in vitro* cleavage assays when compared with the substrates lacking homologous flanking sequences (such as TIRG105) and substrates with AT-rich TSDs (such as TIRG256 and TIRG257). Thus, we conclude that homologous sequences, especially the CG-rich TSD sequences that flank the TIRs, greatly benefit ProtoRAG-mediated recombination, possibly by improving the efficiency of TIR recognition by ProtoRAG.

### Characterization of the essential domains in BbRAG1L responsible for the activity of *ProtoRAG*

**Figure 6.**
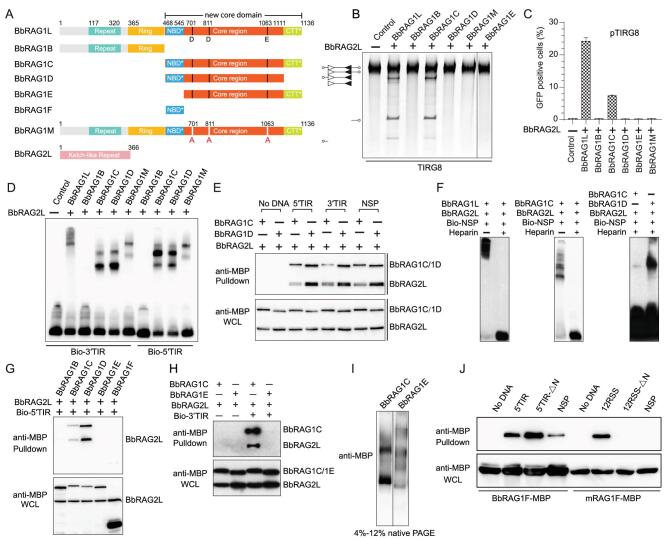
Domains in BbRAG1L essential for the activity of ProtoRAG. (A) Diagram showing the domains of truncated BbRAG1Ls and the active site mutations in BbRAG1L and BbRAG2L. Domains are defined according to the sequence alignment with mouse RAG1 recombinase [20]. (B) Cleavage of the TIRG8 substrate by the indicated BbRAGL proteins *in vitro*. The composition of the cleavage product is shown on the left according to the lengths of the corresponding fragments. Unfilled and filled triangles indicate the 5′TIR and 3′TIR sequences of *ProtoRAG*, respectively. (C) Quantification of the GFP-positive cells produced by BbRAGL-mediated recombination with the pTIRG8 substrate. The composition of the distinct truncated BbRAG1L proteins is shown in (A). (D) EMSA assay to detect the binding of BbRAGL proteins with 5′TIR and 3′TIR. The indicated BbRAGL proteins and their truncated forms were purified. The binding reactions were separated on a 3.5/8% native TBE-PAGE gel. (E) Pull-down assay to detect the binding of BbRAG1C and BbRAG1D with 5′TIR and 3′TIR. Both BbRAG1C and BbRAG1D were co-expressed with BbRAG2L. The NSP probe was mutated from 5′TIR by scrambling the sequences. (F) EMSA assay to detect the binding of BbRAG1L, BbRAG1C and BbRAG1D with nonspecific DNA. The NSP probe was mutated from 5′TIR by scrambling the sequences. (G) Pull-down assay to detect the binding of BbRAG1E and BbRAG1F with 5′TIR. Both BbRAG1E and BbRAG1F were co-expressed with BbRAG2L. WCL, whole-cell lysates. (H) Pull-down assay to detect the binding of BbRAG1E/BbRAG2L with 3′TIR. (I) Electrophoretic separation of singly purified BbRAG1C and BbRAG1E by 4%–12% native PAGE. (J) Comparison of the DNA-binding ability of the NBD* domain of BbRAG1 (BbRAG1F) and the NBD domain of mRAG1 (mRAG1F). The DNA probes are shown as indicated and the TR5 in 5′TIR-ΔN and the nonamer in 12RSS-ΔN were mutated. The NSP probe was mutated from 5′TIR and 12RSS by scrambling the TIR and 12RSS sequences, respectively.

The sequence comparison revealed that both BbRAG1L and BbRAG2L contain a conserved core region similar to that of vertebrate RAG proteins. However, the NBD* and CTT* domains in BbRAG1L are highly divergent from those of its mammalian counterparts. As mentioned above, a pair of unique 5′/3′TIRs was essential for the activity of *ProtoRAG*, to further show how the proteins encoded by *ProtoRAG* (namely, BbRAG1L and BbRAG2L) coordinate with these unique TIRs to mediate efficient recombination, we constructed several truncated mutants of BbRAG1L and BbRAG2L according to a domain architecture comparison with their mammalian counterparts for *ex vivo* recombination efficiency and *in vitro* cleavage assays (Fig. 6A). As shown, in the presence of BbRAG2L, only the full-length sequence and that containing the core region plus the CTT* of BbRAG1L (BbRAG1C) could mediate efficient cleavage *in vitro* and subsequent recombination *ex vivo* (Fig. 6B and C). All other constructs, including the N-terminal construct (BbRAG1B) or the construct with the mutation of the DDE amino acids in BbRAG1L (BbRAG1M) and the deletion of the NBD* domain (BbRAG1E) or CTT* domain (BbRAG1D) from BbRAG1C, abolished the recombination activity (Fig. 6B and C). Moreover, BbRAG1L was more efficient than BbRAG1C in this reaction (Fig. 6C). These observations indicate that not only the NBD* domain, but also the CTT* domain, is essential for the cleavage activity of BbRAG1L, which is different from that observed for the mouse RAG1 (mRAG1) core, which excludes the CTT domain [2]. Moreover, among these truncated or mutated proteins, the deletion of CTT* in BbRAG1D seems to lead to the normal binding of TIRs, as observed for BbRAG1C, except for a small enhancement of DNA binding (Fig. 6D). This observation was reconfirmed by a pull-down assay (Fig. 6E). However, the enhancement of DNA binding seems to be nonspecific because the results for nonspecific DNA were similar (Fig. 6E). To decrease the nonspecific binding of BbRAGL transposases with nonspecific DNAs, heparin was added into the EMSA reaction. Under these conditions, the nonspecific DNA-binding ability of BbRAG1L and BbRAG1C was greatly impaired, but it was clearly shown for BbRAG1D (Fig. 6F). Thus, we assumed that the CTT* domain of BbRAG1L is not only critical for the cleavage activity of BbRAG1L, but also determines the binding specificity of the BbRAG1L/2L complex.

As shown in Fig. 6B and C, the NBD* domain in BbRAG1L is essential for TIR cleavage *in vitro* and recombination *ex vivo*. We then showed that the deletion of NBD* eliminated the interaction of BbRAG with 5′/3′TIRs and that NBD* alone (BbRAG1F) directly interacts with BbRAG1L weakly (Fig. 6G and H). Moreover, in native conditions, the deletion of NBD* (BbRAG1E) led to the formation of fewer stable complexes than BbRAG1C (Fig. 6I), indicating that NBD* is critical for the stabilization of the BbRAGL complex. However, unlike the NBD of mouse RAG1, which can bind with 12RSS specifically, the NBD* of BbRAG1L alone seems to bind DNA nonspecifically (Fig. 6J). In short, the NBD* domain of BbRAG1L may be another DNA-binding domain, although its target element is still unknown. Moreover, the NBD* domain of BbRAG1L contributes significantly to the stabilization of the BbRAGL complex, as does the intertwined NBD domain in the RAG–RSS complex.

## DISCUSSION

Asymmetric 12RSS and 23RSS are critical for orderly V(D)J recombination mediated by RAG recombinase when recombining the D–J and V–DJ elements step by step. The discovery of *ProtoRAG* in amphioxus provided critical proof that vertebrate RAG recombinases originated from an ancient RAG transposon [20], but the divergence of the TIRs of *ProtoRAG* and other *RAG-like* transposons with vertebrate RSSs makes it difficult to determine whether or how 12/23RSS persisted in the vertebrate RAG system that evolved from paired TIRs in ancient RAG transposons (Supplementary Fig. 1). Here, we elucidate the functional requirements of TIRs to induce efficient *ProtoRAG* activity, which may have important implications for the early evolution of vertebrate VDJ recombination.

### Asymmetrical TIRs with bipartite conserved elements are required for efficient *ProtoRAG* activity

The conserved nucleotides in TIRs of transposons and RSSs involved in V(D)J recombination have always correlated with their essential roles in transposition and recombination, respectively [7,29,30]. Through the alignment of several copies of *ProtoRAG* TIRs from three species of lancelets, we defined the conceptual structure of the typical TIRs of *ProtoRAG*, which are composed of conserved TR1 and TR5 elements and partially conserved TRsp elements with lengths of 27 bp in 5′TIR or 31 bp in 3′TIR that separate them. The conserved TR1 element shows great resemblance to the consensus RSS heptamer, especially for their identical first CAC triplet. CAC is particularly important for TIR cleavage, which is correlated with the presence of the highly conserved contacting amino acids in BbRAG1L transposase. In addition, conserved CAC and heptamer-like sequences can also be found in the predicted TIRs of other RAG-like transposons in deuterostomes (Supplementary Fig. 1), so the recognition of the heptamer-like sequence and the nicking of the ‘CAC’ triplet in TIRs are likely to comprise an ancient and conserved mechanism shared by all RAG and RAG-like transposons. The other conserved TR5 element shows little sequence similarity with the RSS nonamer. The deletion and mutation of TR5 seemed to have no or little effect on the substrate cleavage efficiency of ProtoRAG *in vitro* (Fig. 1E and 3C) [21], but TR5 mutations obviously decreased the recombination efficiency and the precise cleavage of TIRs by ProtoRAG *ex vivo* (Fig. 3E). Consistently, the partial deletion of TRsp, which was equivalent to the mutation of the TRsp and TR5 elements at the same time, also caused a dramatic decrease in the recombination efficiency and generated specific amounts of imprecise cleavage products. Thus, TR5 may function as another important element in *ProtoRAG* to facilitate the precise cleavage of TIRs *ex vivo*. Recently, the structures of the BbRAG–TIR complex were determined, but no density for NBD* and the TR1-distal 25-bp sequence was discernible [21]. By comparison, RSS nonamers were anchored by the NBD domain of RAG1 in the RAG–RSS complex and play critical roles in the activity of RAG recombinase. Thus, whether the TR5 element can be directly contacted by the BbRAG complex needs to be further determined.

As linkers between TR1 and TR5, the 5′TRsp and 3′TRsp of the 5′/3′TIR in *ProtoRAG* from *B. belcheri* have rigid lengths and an overall identity of 45.5% according to manual alignment. However, except for some critical nucleotides in TRsp, most of the nucleotides in TRsp comprised low-level variations. In addition, ProtoRAG prefers the asymmetric 5′/3′TIR, which results in more efficient activity than the symmetric pairs of TIRs. These results indicate that 5′TIR and 3′TIR are asymmetric not only in their sequences, but also in their function during BbRAG-mediated recombination. Interestingly, putative TIRs in other RAG-like transposons appeared to contain distinct conserved elements in their 3′termini when forming asymmetric TIRs (Supplementary Fig. 1), like the situations in *ProtoRAG* TIRs and RSS. Thus, in addition to the conserved nucleotides in TIRs, the preference for asymmetric TIRs might be a common feature shared by ancient RAG transposons, which supports their common origin from an ancestral RAG transposon with asymmetric TIRs.

### Diversification of BbRAG transposase and its target preferences

To coordinate with diversified TIRs, BbRAG transposase has developed new features in addition to its core region, which is homologous with that of vertebrate RAG proteins. First, we found that, in addition to the conserved core domain corresponding to its vertebrate counterpart, the new CTT* domain was found to be essential for the cleavage activity of BbRAG1L and helpful for the specific binding of BbRAG1L to TIRs. In the recently published crystal structure of the BbRAG–TIR complex, CTT* may function as a new DNA-binding domain to interact with the TR2 region [21]. Thus, we suggest that the functional core domain in BbRAG1L should include the conserved core domain and the new CTT* domain. Second, like the NBD domain of the vertebrate RAG1 protein, which intertwines with itself to form a dimer [26], the NBD* domain in BbRAG1L contributes to the stabilization of the BbRAGL–TIR complex. However, unlike the NBD of mouse RAG1, which can bind with 12RSS specifically, the NBD* of BbRAG1L alone seems to bind DNA nonspecifically (Fig. 6J). Notably, the density of the NBD* domain is absent from the structure of the BbRAG–TIR complex [21]; thus, whether NBD* may bind TR5 as does the NBD of mouse RAG1 to interact with the nonamer needs to be further determined. Here, we have determined that the NBD* domain of BbRAG1L may be another DNA-binding domain and contribute to the stabilization of the BbRAGL complex as the intertwining NBD domain does in the RAG–RSS complex.

TSD sequences are an important sign of transposition and always surround the TIRs outside of the transposon. Here, we also showed that BbRAGL-mediated recombination could be enhanced by homologous flanking sequences, especially CG-rich TSD sequences. However, the preference for homologous flanking sequences was not observed for mouse RAG-mediated recombination [28], although the coding sequences have been shown to be involved in the interaction between RSS and RAG proteins [4]. In vertebrates, there are a total of several hundred V, D and J elements in the genomic regions of *BCR* and *TCR*, so RAG recombinases need to recognize the RSSs surrounding the randomly selected V, D and J elements to induce diversified recombination. However, during the transposition of *ProtoRAG*, BbRAG transposases must specifically recognize the paired TIRs in a single *ProtoRAG* copy to avoid confusing it with other TIRs in different *ProtoRAG* copies in the genome. Thus, the requirement for homologous sequences that flank the TIRs may help the BbRAG transposases to accurately target the paired TIRs of *ProtoRAG*, which is advantageous for the survival of the transposon. After the RAG transposase evolved into RAG recombinase, such a preference was lost, which was advantageous for the random recombination of V, D and J elements in the host.

In summary, we revealed that, similarly to that of RSS during RAG-mediated recombination, the activity of *ProtoRAG* is highly dependent on the coordination between BbRAGL transposase and its asymmetric 5′TIR and 3′TIR. Such functional coordination suggests that the preference for asymmetric TIRs composed of bipartite conserved elements and a separating element with a distinct length may be shared by the common ancestor, which has important implications for the early evolution of vertebrate VDJ recombination.

## MATERIALS AND METHODS

### Generation of TIR-altered vectors

The pTIRG8 vector was constructed in our previous study. An inverted pair of *ProtoRAG* 5′-TIR and 3′-TIR sequences separated by a transcriptional stop sequence were inserted into a reporter plasmid between the promoter and a GFP gene to form a fluorescence reporter plasmid. The TIR-altered vectors were produced by the Quick Change Lightning Multi Site-Directed Mutagenesis Kit with appropriately designed primers. The altered regions were confirmed by sequencing.

### Recombination assay to detect GFP-positive cells

HEK293T cells were transfected with 0.4 μg of *BbRAG1L* and *BbRAG2L* expression plasmids and 0.4 μg of substrate plasmids (pTIRGs) as indicated with jetPrime (PolyPlus transfection) according to the manufacturer's instructions. After 48 h, the cells were treated with trypsin, resuspended in DMEM and centrifuged for 5 min at 800 *g*. The cell pellets were washed twice with PBS and resuspended in PBS at a density of ∼10^6^ cells/ml. The GFP-positive cells were then analysed by flow cytometry (Beckman CytoFLEX). For all the recombination assays in HEK293T cells, the results were obtained from at least three independent experiments, and the values are expressed as the mean (+/– SEM). Data were analysed using two-tailed Student's *t*-test for unpaired variables when appropriate. The three significance levels were defined as usual according to different *p*-values as follows: *: *p* < 0.05, **: *p* < 0.01, ***: *p* < 0.001.

### Analysis of HDJs in recombinant products

After the analysis of GFP-positive cells through flow cytometry, the plasmid DNA was recovered from the transfected HEK293T cells by alkaline lysis. The recovered plasmids were first treated with BstXI and BsrGI endonucleases (NEB) to cleave the original substrates, which would preserve only the recombinant substrates with intact HDJ sequences. The reaction samples were purified with a PCR purification kit (QIAGEN) and subjected to PCR amplification (35 cycles). The specific PCR primers used were pTIR-P1 and pTIR-P2 (Supplementary Table 1). The PCR products were separated on 2% agarose gels. The HDJ DNA was extracted with a Gel Extraction kit (QIAGEN) and ligated into the pGEM-T Easy vector (Promega), which was then transformed into *Escherichia coli* DH5α for Sanger sequencing. The HDJ sequences were aligned with ClustalX and manually modified with Genedoc.

### Protein expression and purification for *in vitro* analysis

The codons of the *BbRAG1L* and *BbRAG2L* coding sequences were optimized for efficient expression in human cells, and the gene fragments were cloned into the pTT5 vector to produce a fusion protein with a maltose-binding protein (MBP) tag at its N-terminus. BbRAG1L and BbRAG2L were separately transfected or co-transfected into HEK293T cells using PEI. Cells were harvested 48 h after transfection. The harvested cells were resuspended in lysis buffer (25 mM Tris (pH 7.5), 500 mM NaCl and 1 mM DTT). The proteinase cocktail (Roche) was added as instructed. The resuspended cells were sonicated for a sufficient amount of time to disrupt the cells and centrifuged at 15 000 rpm for 30 min. The supernatant was filtered and the cleared lysate was mixed with pre-equilibrated amylose resin (NEB) for 1 h at 4°C with shaking. The resin was washed with 10 volumes of lysis buffer and the proteins were eluted with elution buffer (25 mM Tris (pH 7.5), 0.5 M KCl, 1 mM DTT and 10 mM maltose). The eluate was concentrated and dialysed in dialysis buffer (25 mM Tris, pH 7.5, 150 mM KCl, 2 mM DTT and 10% glycerol) with an Amicon Ultra-4 centrifugal filter (Millipore) at 4°C. The dialysed protein samples were frozen in small aliquots and stored at −80°C. The protein concentration was determined by the Bradford method by comparison to a BSA standard curve. The other truncated or mutated BbRAG1L proteins were expressed and purified according to a similar procedure.

### *In vitro* cleavage assay

The TIR cleavage substrates were generated by PCR by using the corresponding substrate plasmids as templates and then purified from agarose gels. These substrates contained identical sequences except for the indicated alterations. The primers used for these PCRs were TIRG_SUB_U1 and TIRG_SUB_L1. The 16-μl cleavage reactions contained 25 nM of the co-expressed MBP-BbRAG1L/2L proteins (monomeric BbRAG1L/BbRAG2L concentration), 175 ng HMGB1 and 10 nM substrate DNA in reaction buffer (25 mM MOPS (pH 7.0), 50 mM KCl, 2 mM DTT and 1.5 mM MgCl2) and were incubated at 37°C for 1 h. The reactions were stopped by adding 1.25 μl 2.5% SDS, 5 μl proteinase K (150 μg/ml) and 2 μl 0.5 M EDTA and incubated at 55°C for 1 h. They were then mixed with 80% glycerol before being loaded on 6% native TBE (Tris-borate-EDTA) acrylamide gels. After electrophoresis, the gels were stained with SYBR GOLD (Invitrogen) and imaged using a G-BOX (SynGene).

### EMSA

The basic binding assay mixtures (10 μl) contained 20 fmol biotin-labeled oligonucleotide substrate DNA in 25 mM MOPS (pH 7.0), 50 mM KCl, 2 mM DTT, 1.5 mM CaCl_2_, 2.5% glycerol, 50 nM HMGB1 and 100 nM single-stranded nonspecific oligonucleotides. BbRAG1L and BbRAG2L were added as indicated and the concentration of each RAG protein was approximately 50 nM. The reactions were incubated at 25°C for 20 min. After incubation, 2.5 μl 5 × gel loading buffer was added to each reaction and the samples were analysed by 3.5%/8% TBE gels. The biotin-DNAs in the gel were transferred to a nylon membrane and blotted as instructed by the Pierce EMSA LightShift Chemiluminescent EMSA Kit (Thermo Scientific). The sequences of the oligonucleotides used in this study are listed in the Supplementary Table 1. The biotin-labeled double-stranded DNA probes were produced by annealing the biotin-labeled forward primers with the reverse primers. The competitive probes were produced by annealing the unlabeled forward primers with the reverse primers.

### Pull-down of RAG proteins with biotin-labeled DNA

The plasmids expressing BbRAGL or RAG proteins were transfected into HEK293T cells. After 48 h, the harvested cells were resuspended in lysis buffer (50 mM Tris (pH 7.5), 150 mM NaCl, 0.5% NP40, 5% glycerol, 1.5 mM CaCl_2_ and proteinase inhibitor) for 30 min and then centrifuged at 15 000 rpm for 10 min to separate the supernatant. The biotin-labeled DNA primers were annealed in a PCR amplifier and added to the supernatant, after which they were allowed to bind with the proteins for 1 h at 4°C. The activated streptavidin agarose resin (Invitrogen) was added to the DNA–protein mixtures and allowed to bind with the biotin–DNA for 40 min at 4°C, and the resin was then centrifuged at 800 *g* for 5 min. The harvested resin was washed three times with lysis buffer and then denatured at 100°C in protein loading buffer. In the end, the prepared samples were subjected to normal Western blotting to detect the pulled-down proteins.

## Supplementary Material

nwz179_Supplemental_File
