# Supplementary material for: Functional requirement of terminal inverted repeats for efficient ProtoRAG activity reveals the early evolution of V(D)J recombination
Source: Natl Sci Rev. 2019 Nov 13;7(2):403–17. doi: 10.1093/nsr/nwz179 (PMC8289069; doi:10.1093/nsr/nwz179)
Supplement: nwz179_Supplemental_File [file nwz179_supplemental_file.pdf]

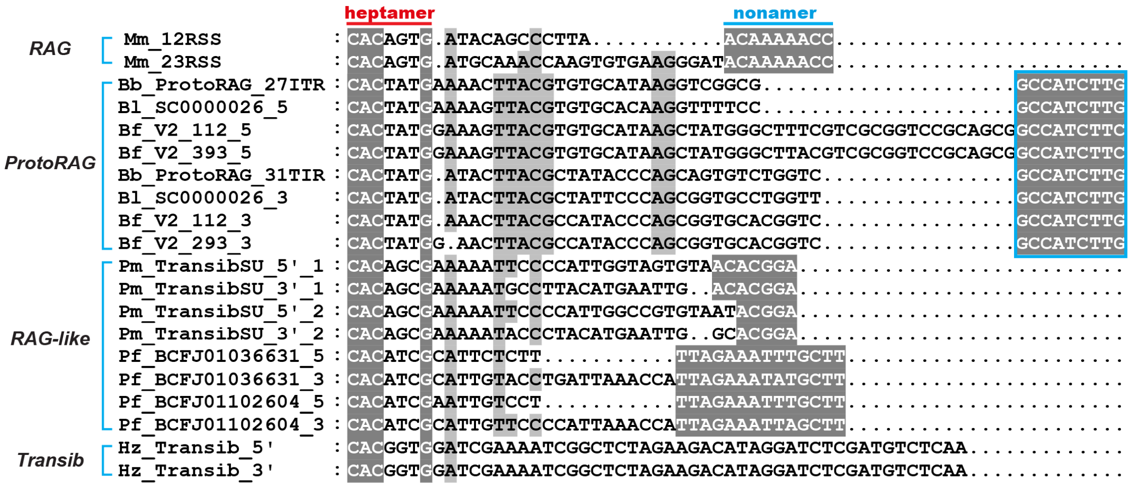

Supplementary Figure 1. Sequences comparison of RSSs and TIRs of several RAG-like transposons.

The RSS and TIR sequences were obtained from public database, including the 12RSS and 23RSS [1], 5'TIR and 3'TIR of *Proto*RAG in amphioxus [2-4], 5'- and 3'-TIR of RAG-like transposon in 5'- and 3'-TIR of *TransibSU_PM* [5], 5'- and 3'-TIR of *Hz_Transib* [6]. The heptamer and nonamer were marked with red and blue color, respectively. Abbreviation of species name, Bb: *Branchiostoma belcheri*; Bl: *Branchiostoma lanceolatum*; Bf: *Branchiostoma floridae*; Pm: *Patiria miniata*; Pf: *Ptychodera flava*; Hz: *Helicoverpa zea*.

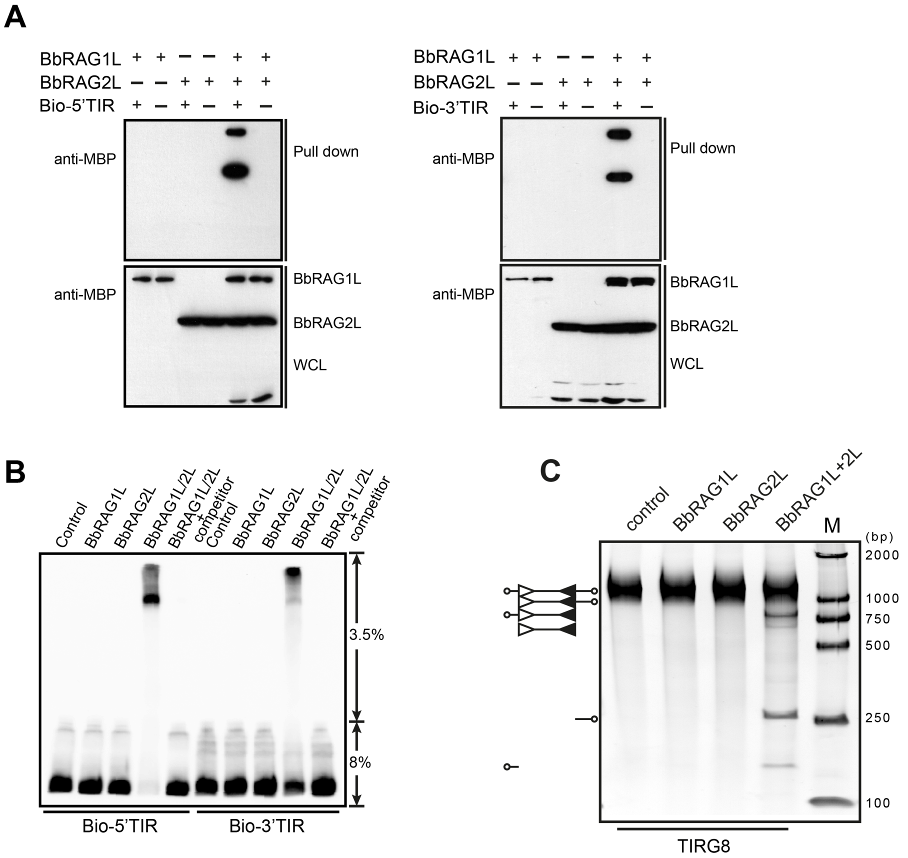

Supplementary Figure 2. TIRs binding and substrates cleavage depend on the cooperation of BbRAG1L and BbRAG2L.

(A) Pull down assay to detect the binding of BbRAG1L and BbRAG2L with 5’TIR and 3’TIR.

(B) EMSA assay to detect the binding of BbRAG1L and BbRAG2L to biotin-5’TIR and biotin-3’TIR. We used 200-fold unlabeled 5’TIR or 3’TIR as a competitive probe, and binding reactions were separated on a 3.5/8% native TBE page gel.

(C) Cleavage of TIRG8 substrate by indicated proteins. The composition of the cleavage product was shown on the left according to the length of corresponding fragments.

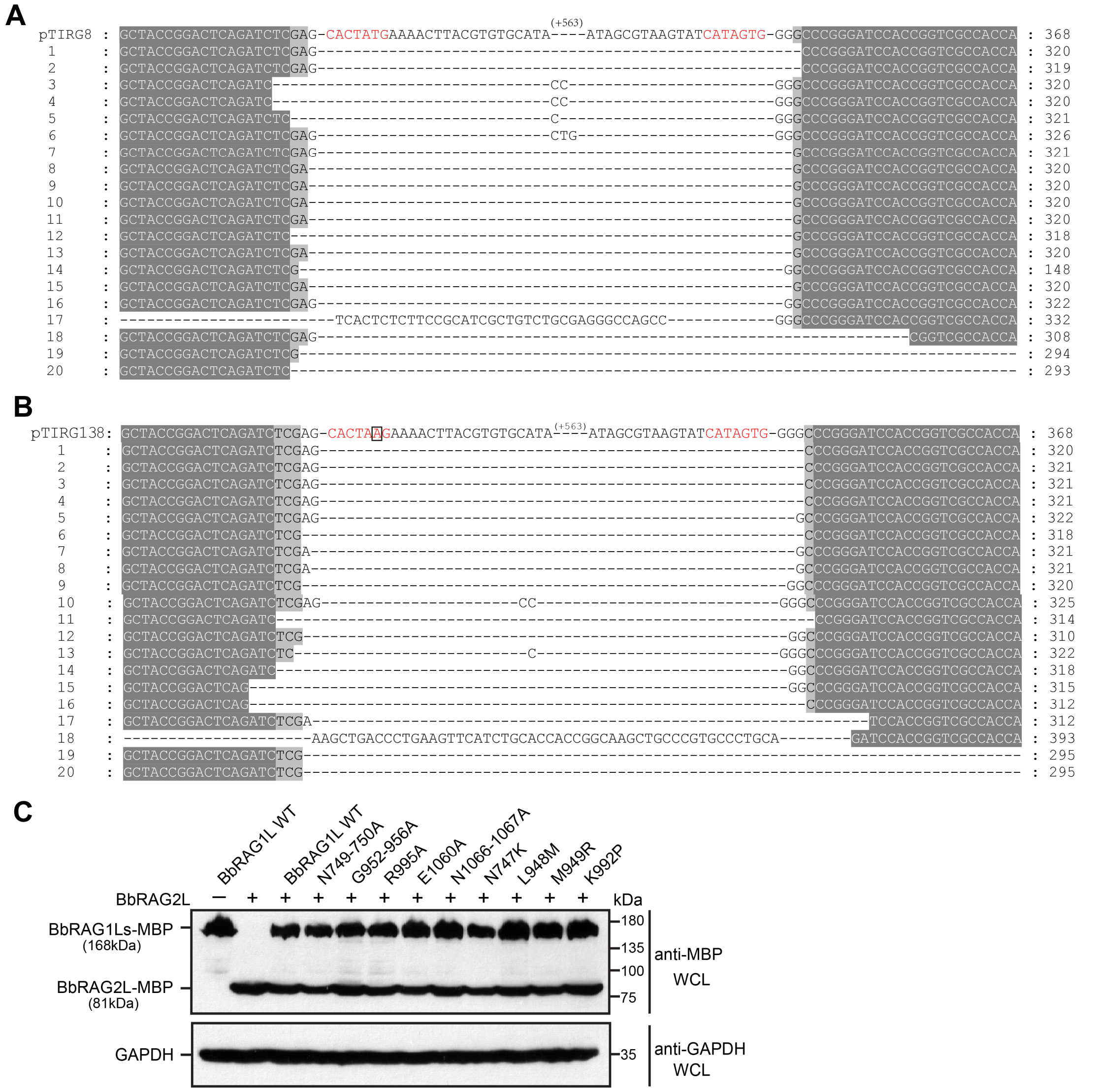

Supplementary Figure 3. Analysis of HDJ products produced by BbRAG1L/2L mediated recombination and expression of BbRAG1L mutants with BbRAG2L

(A) Sequence alignment of HDJ products from recombination of pTIRG8 by BbRAG1L/2L.

(B) Sequence alignment of HDJ products from recombination of pTIRG138 by BbRAG1L/2L. The first line shows partial sequence of the un-cleaved pTIRG8 (A) and pTIRG138 (B). The TR1 in 5'/3'TIR were marked with red color, and the mutated nucleotides were marked with unfilled rectangle. The HDJ products from 20 clones of E. coli were sequenced, and their sequences were aligned with that of un-cleaved substrates.

(C) Co-expression of BbRAG1L mutants with BbRAG2L in HEK293T cells. Name of the BbRAG1L mutants shown their mutated amino acids, such as, N749-750A: amino acids in position 749 and 750 were both mutated into A. All BbRAG proteins were fused with MBP tag at their N-terminus. Whole cell lysis (WCL) was used for western blot to detect the expression of BbRAG1L mutants and BbRAG2L, and the total proteins were quantified with GAPDH.

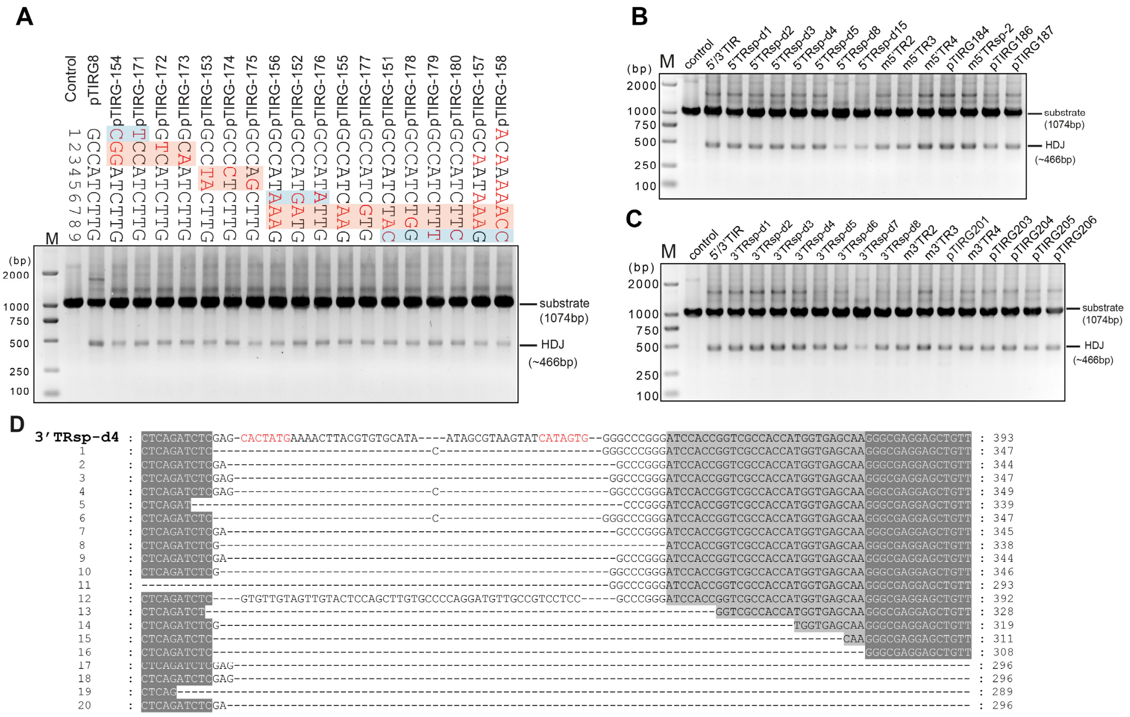

Supplementary Figure 4. PCR assay to detect recombined HDJ products produced during BbRAG1L/2L-mediated recombination with the corresponding substrates.

(A) Detection of recombined HDJ products produced during BbRAG1L/2L-mediated recombination with the TR5-mutated TIR substrates.

(B) Detection of recombined HDJ products produced during BbRAG1L/2L-mediated recombination with the 5'TRsp-deletion and 5'TRsp-mutation substrates.

(C) Detection of recombined HDJ products produced during BbRAG1L/2L-mediated recombination with the 3'TRsp-deletion and 3'TRsp-mutation substrates.

(D) Alignment of the HDJ sequences produced in recombination of 3'TRsp-d4 substrate. First line is the original sequence of 3'TRsp-d4, with the TR1 colored with red. HDJ sequences from 20 clones were analyzed. Cleavage on the plasmid backbone caused long nucleotides deletion to be marked with dash.

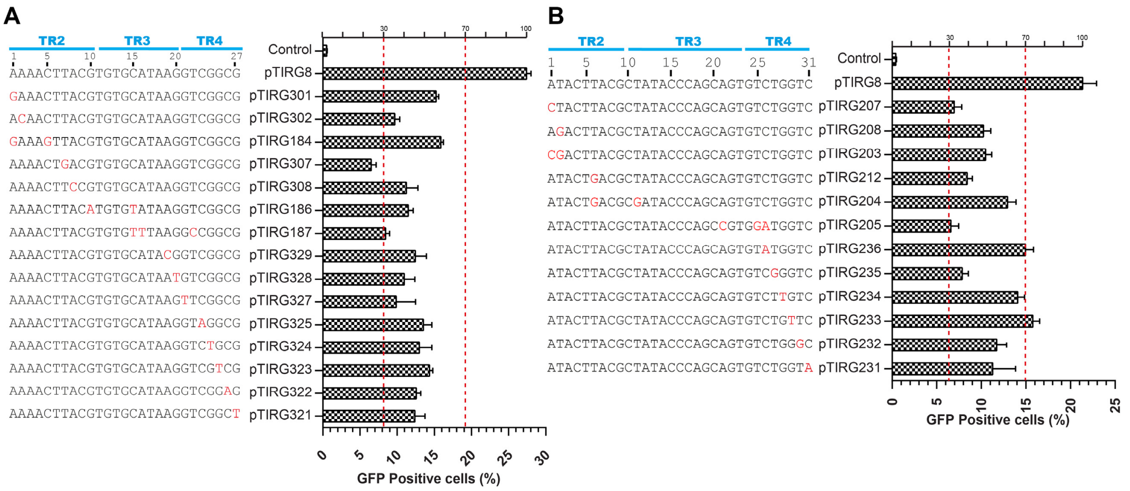

Supplementary Figure 5. Quantification of GFP positive cells produced by BbRAG1L/2L mediated recombination with the 5'TRsp-mutation TIR substrates (A) and 3'TRsp-mutation TIR substrates (B).

Position and definition of regions are consistent with Fig 4. The substitution of nucleotides was shown as indicated (marked with red color). Y axis refers to the TIR substrates. Bottom X axis showed the percentage of GFP positive cells, and upper X axis showed the percentage of GFP positive cells relative to the value of pTIRG8. Three levels of effect (slightly, moderately, dramatically) were defined according to the value on the right Y axis (>70%, 30%-70%, <30%). Results are expressed as means (+/-SEM).

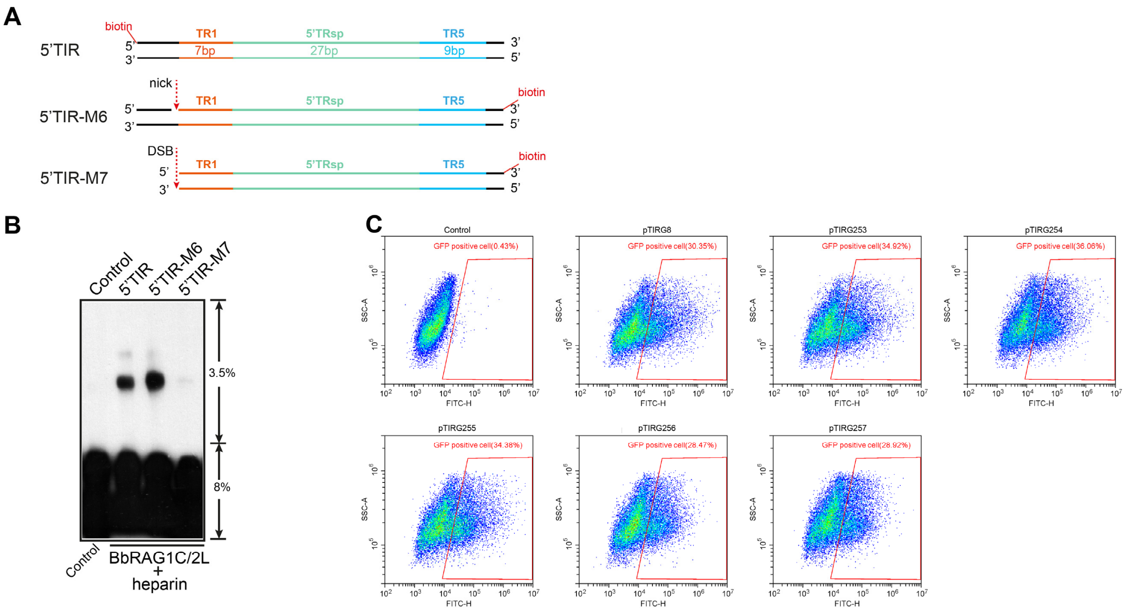

Supplementary Figure 6. Flanking sequences are important for the activity of BbRAG1L/2L.

(A) Diagram of modified 5'TIRs to contain a nick or broken flanking sequence aside of the heptamer.

(B) EMSA assay to detect the binding of BbRAG1C/2L with nicked 5'TIR and broken 5'TIR.

(C) Quantification of GFP-positive cells produced by BbRAG1L/2L-mediated recombination with TSD sequences containing TIR substrates. One of the representative results from three independent experiments were shown. The TSD sequence of these substrates were shown in Fig 5C.

Supplementary Table 1. Oligonucleotides used in PCR and EMSA assays

Name	Sequence
BIO-3’TIR-F	Biotin-5’GGTGGATCCCGGGCCCCACTATGATACTTACGCTATACCCAGCAGTGTCTGGTCGCCATCTTGTAAGAT3’
3’TIR-R	5’ATCTTACAAGATGGCGACCAGACACTGCTGGGTATAGCGTAAGTATCATAGTGGGGCCCGGGATCCACC3’
BIO-5’TIR-F	Biotin-5’CGGACTCAGATCTCGAGCACTATGAAAACTTACGTGTGCATAAGGTCGGCGGCCATCTTGGTAATG3’
5’TIR-R	5’CATTACCAAGATGGCCGCCGACCTTATGCACACGTAAGTTTTCATAGTGCTCGAGATCTGAGTCCG3’
5’TIR-MF6	5’CGGACTCAGATCTCGAG3’
BIO-5’TIR-MF7	5’CACTATGAAAACTTACGTGTGCATAAGGTCGGCGGCCATCTTGGTAATG3’-Biotin
5’TIR-MR7	5’CATTACCAAGATGGCCGCCGACCTTATGCACACGTAAGTTTTCATAGTG3’
Bio-NSP-F	Biotin-5’CGGACTCAGATCTCGAGTCCAGAAGTCGCTCGACTGTGGTTTAATCGCTTGTAGCGTGGTCTGGTAATG3’
NSP-R	5’CATTACCAGACCACGCTACAAGCGATTAAACCACAGTCGAGCGACTTCTGGACTCGAGATCTGAGTCCG3’
BIO-5’TIR-△N-F	Biotin-5’CGGACTCAGATCTCGAGCACTATGAAAACTTACGTGTGCATAAGGTCGGCGGTAATG3’
5’TIR-△N-R	5’CATTACCGCCGACCTTATGCACACGTAAGTTTTCATAGTGCTCGAGATCTGAGTCCG3’
Bio-12RSS-F	Biotin-5’GATCTGGCCTGTCTTACACAGTGATACAGACCTTAACAAAAACCCTGCAG 3’
12RSS-R	5’CTGCAGGGTTTTTGTTAAGGTCTGTATCACTGTGTAAGACAGGCCAGATC 3’
Bio-12RSS-△N-F	Biotin-5’GATCTGGCCTGTCTTACACAGTGATACAGACCTTACTCTGGCTGCTGCAG 3’
12RSS-△N-R	5’CTGCAGCAGCCAGAGTAAGGTCTGTATCACTGTGTAAGACAGGCCAGATC 3’
Bio-RNSP-F	Biotin-5’GATCTGGCCTGTCTTAGGTCAATGCTGTAGAACTCGTCCTGTACCTGCAG 3’
RNSP-R	5’CTGCAGGTACAGGACGAGTTCTACAGCATTGACCTAAGACAGGCCAGATC 3’
TIRG_SUB_U1	5’TTTGGCACCAAAATCAACGG3’
TIRG_SUB_L1	5’GGACTTGAAGAAGTCGTGCT3’
pTIR-P1	5’ACTTGGCAGTACATCTAC3’
pTIR-P2	5’GATGAACTTCAGGGTCAG3’

References

1. Swanson PC. The bounty of RAGs: recombination signal complexes and reaction outcomes. *Immunol Rev*. 2004; **200**: 90-114.
2. Huang S, Tao X, Yuan S *et al.* Discovery of an Active RAG Transposon Illuminates the Origins of V(D)J Recombination. *Cell*. 2016; **166**: 102-14.
3. Marletaz F, Firbas PN, Maeso I *et al.* Amphioxus functional genomics and the origins of vertebrate gene regulation. *Nature*. 2018; **564**: 64-70.
4. Putnam NH, Butts T, Ferrier DE *et al.* The amphioxus genome and the evolution of the chordate karyotype. *Nature*. 2008; **453**: 1064-71.
5. Kapitonov VV, Koonin EV. Evolution of the RAG1-RAG2 locus: both proteins came from the same transposon. *Biol Direct*. 2015; **10**: 20.
6. Hencken CG, Li X, Craig NL. Functional characterization of an active Rag-like transposase. *Nat Struct Mol Biol*. 2012; **19**: 834-6.
